# Distribution of Antisense Oligonucleotides in Rat Eyeballs Using MALDI Imaging Mass Spectrometry

**DOI:** 10.5702/massspectrometry.A0070

**Published:** 2018-09-11

**Authors:** Yuko Nakashima, Mitsutoshi Setou

**Affiliations:** 1 International Mass Imaging Center and Department of Cellular and Molecular Anatomy, Hamamatsu University School of Medicine, Japan; 2 Preeminent Medical Photonics Education & Research Center, Japan; 3 Department of Anatomy, The University of Hong Kong, China

**Keywords:** MALDI-IMS, antisense oligonucleotide, distribution, rat eyeball, oligonucleotide therapeutics

## Abstract

Oligonucleotide-based therapeutics such as antisense oligonucleotides, small interfering RNAs (siRNAs), decoy and aptamer have been extensively developed. To investigate the pharmacokinetics of oligonucleotide therapeutics, it is common to label a radioisotope in a nucleic acid and visualize it. However, if the labeled terminal nucleotide is decomposed by a nuclease *in vivo*, only the labeled nucleotide is detected, and it is impossible to observe the nucleic acid exhibiting the drug effect. The distribution of biomolecules, such as phospholipids, proteins, and glycolipids, can be obtained and visualized without labeling using matrix-assisted laser desorption/ionization imaging mass spectrometry (MALDI-IMS). MALDI-IMS is also used in pharmacokinetic analysis to visualize a parent drug and its metabolites simultaneously. In this study, we reported a methodology for oligonucleotides analysis by MALDI-IMS. When phosphorothioate antisense oligonucleotide was administered into the eyeball of rats, it reached the retina after 30 min without undergoing decomposition by nucleases.

## INTRODUCTION

Oligonucleotide-based therapeutics such as antisense oligonucleotides,^1–3)^ small interfering RNAs (siRNAs),^4–6)^ decoy^7)^ and aptamer^8)^ have been developed extensively. Oligonucleotide have a low stability against nuclease *in vivo*, but owing to the remarkable progress of chemically modified nucleic acids^2,9–11)^ and drug delivery system technology,^12,13)^ many stable and highly effective candidate products have been developed. Although only six oligonucleotide therapeutics have been approved to date, there are about 20 candidate products in Phase III or higher stages.^14)^ To investigate the pharmacokinetics of oligonucleotide therapeutics, it is common to label a radioisotope in a nucleic acid and visualize it. However, during this process if the labeled terminal nucleotide is decomposed by a nuclease *in vivo*, only the labeled nucleotide is detected, and it is impossible to observe the nucleic acid exhibiting the drug effect. Moreover, the labeling method is costly and labor intensive, and it evokes radioactivity-related safety issues.

Matrix-assisted laser desorption/ionization imaging mass spectrometry (MALDI-IMS) has been developed to reveal the distribution of biomolecules, such as phospholipids,^15–20)^ proteins,^21–23)^ and glycolipids.^24,25)^ In fact, MALDI-IMS analysis can be used to visualize the distribution of biomolecules without any labeling. MALDI-IMS is also used for pharmacokinetic analyses, to visualize a parent drug and its metabolites simultaneously.^26–28)^

Through the development of the MALDI method, mass spectrometry has been widely used as a means of qualitative analysis of chemically synthesized oligonucleotides.^29–31)^ However, in the only IMS study of nucleic acids, stable isotope-labeled nucleic acids were detected by secondary ion mass spectrometry (SIMS)-IMS.^32,33)^ *Via* MALDI-IMS, even when the nucleotides are decomposed by nucleases *in vivo*, decomposed products can be detected. Developing a method for detecting oligonucleotides by MALDI-IMS should greatly contribute to the progress of nucleic acid research.

In the present study, we focused on antisense oligonucleotides that are the most developed among oligonucleotide therapeutics.^1–3)^ We describe the development of a new methodology for oligonucleotide analyses by MALDI-IMS. First, the limit-of-detection (LOD) of oligonucleotides in the MALDI-IMS method was examined using phosphorothioate antisense oligonucleotide (ASO-1, ASO-2). Subsequently, we optimized the method of MALDI-IMS analysis of oligonucleotides using tissue samples, which were prepared by administering ASO-1 to mouse thigh muscles. Finally, the antisense oligonucleotide of cytomegalovirus retinopathy (ASO-2), originally approved as an oligonucleotide therapeutic, was locally administered to the vitreous body of rats. Eyeballs were removed 30 min post-administration, followed by MALDI-IMS analysis.

## MATERIALS AND METHODS

### Chemicals

Phosphorothioate oligonucleotides (ASO-1, ASO-2) were obtained from Fasmac Co., Ltd. (Kanagawa, Japan). ASO-1 is composed of 13 nucleotide units “5′-GCA TTG GTA TTC A-3′” (MW 4157.35, EM 4254.42) and ASO-2 is composed of 21 nucleotide units “5′-GCG TTT GCT CTT CTT CTT GCG-3′” (MW 6682.35, EM 6677.59). 3-Hydroxypicolinic acid (HPA) was obtained from Sigma-Aldrich (St. Louis, MO, USA). Ethanol, chloroform, acetic acid, acetonitrile and diammonium hydrogencitrate were purchased from Wako Pure Chemical Industries, Ltd. (Osaka, Japan). Oligonucleotide calibration standard (#206200) was obtained from Bruker Daltonics (Leipzig, Germany) and hydrochloric acid medetomidine was from Nippon Zenyaku Kogyo Co., Ltd. (Tokyo, Japan). Midazolam was purchased from Sandoz K.K. (Tokyo, Japan) and butorphanol tartrate from Meiji Seika Pharma Co., Ltd. (Tokyo, Japan). Eight-week-old male C57BL/6J mice and eight-week-old male SD rats were purchased from SLC (Hamamatsu, Japan).

### Sample preparation for LOD determination in MALDI-IMS

ASO-1 and ASO-2 were prepared in concentrations of 50, 5, 0.5, and 0.05 μM in ultrapure water. A 0.5 μL solution was deposited on an indium-tin-oxide (ITO)-coated glass slide; accordingly, the final volumes of the droplets were 25, 2.5, 0.25, and 0.025 pmol, respectively. 0.5 μL HPA matrix solution (0.2 M HPA, 0.04 M ammonium citrate dibasic, in 50% acetonitrile) was dropped onto the samples. Then, ASO-1 and ASO-2 droplets were subjected to MALDI-IMS (*n*=3).

### Antisense oligonucleotide (ASO-1) administration to mice

All experiments on mice were conducted according to protocols approved by the Animal Care and Use Committee of the Hamamatsu School of Medicine. Eight-week-old male C57BL/6J mice were sacrificed and thigh muscles were extracted. The tissues were injected with ASO-1 (2.5 mM 20 μL or 250 μM 20 μL, in saline, *n*=3) and the muscles were immediately frozen in powdered dry ice and stored at −80°C until use.

### Antisense oligonucleotide (ASO-2) administration to rats

All experiments on rats were conducted according to protocols approved by the Animal Care and Use Committee of the Hamamatsu School of Medicine. Eight-week-old male SD rats were anesthetized using hydrochloric acid medetomidine, midazolam and butorphanol tartrate and then intravitreously injected with ASO-2 (500 μg, in saline). Rats were sacrificed immediately and 30 min after ASO-2 administration (*n*=3 at each time points). The eyes were extracted, embedded in carboxymethyl cellulose, immediately frozen with dry ice/acetone, and stored at −80°C until use.

### Sample preparation of tissue sections for IMS analyses

Before sectioning, the frozen samples were kept for 30 min at −25°C. Tissues blocks were sectioned at −25°C using a cryostat (CM 3050; Leica, Germany) to a thickness of 10 μm. The frozen sections were thaw-mounted on ITO-coated glass slides (Matsunami Glass Ind., Ltd., Japan). Slides containing the tissue sections were washed to remove salts and lipids as previously described. Brieﬂy, the wash protocol comprised 70% ethanol (30 s), 100% ethanol (30 s), Carnoy’s solution (6 : 3 : 1 ethanol : chloroform : acetic acid, v/v/v, 2 min), 100% ethanol (30 s).

### Imaging mass spectrometry

The sections were prepared by spraying HPA matrix solution (5 mg/mL HPA, 0.04 M ammonium citrate dibasic, in 80% acetonitrile, 5 mL) using Image Prep (Bruker Daltonics). MALDI-IMS analyses were performed by a TOF type mass spectrometer (Rapiflex; Bruker Daltonics) in negative-ion and linear modes. The analyzer was calibrated using an oligonucleotide calibration standard (#206200) (Oligo 12 ([M−H]^−^, *m*/*z* 3644.4), Oligo 20 ([M−H]^−^, *m*/*z* 6116.0) and Oligo 30 ([M−H]^−^, *m*/*z* 9190.0)). IMS analyses were performed with a scan pitch of 150 μm.

### Image reconstruction

The resulting ion images were visualized using FlexImaging 4.1 (Bruker Daltonics). Reconstructed ion images were normalized using total ion current and performed baseline subtraction.

## RESULTS

### LOD of oligonucleotide

**Figure 1:**
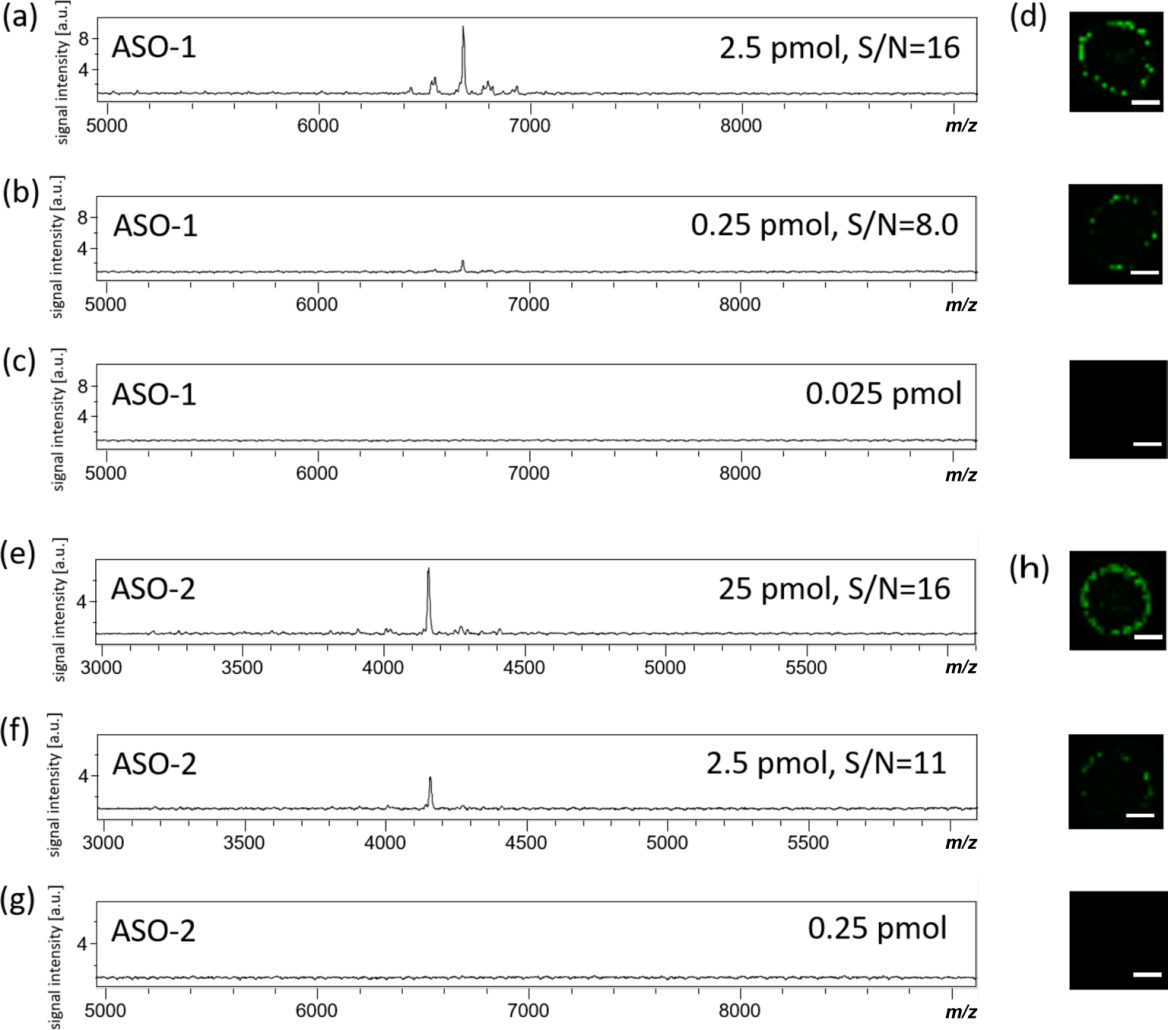
Fig. 1. Limit-of-detection (LOD) determination for antisense oligonucleotide (ASO-1).

In this study, oligonucleotides of two types of sequences (ASO-1 for mice, ASO-2 for rats) were synthesized (Table 1). All phosphodiester bonds were phosphorothioated to increase the resistance to nucleases. First, we investigated LOD of oligonucleotides using MALDI-IMS. For this purpose, three different volumes of ASO-1 and ASO-2 were deposited on each well of an ITO-coated glass slide. In the results of MALDI-IMS analyses, a peak at *m*/*z* 4155 corresponding to the [M−H]^−^ of ASO-1 was detected at 2.5 pmol (Fig. 1a) and 0.25 pmol (Fig. 1b). The LOD value was determined at a signal-to-noise (S/N) ratio of 3. The S/N ratios were acquired by calculating the ratio using the signal intensity of the peak at *m*/*z* 4155 and the maximum signal intensity around the peak at *m*/*z* 4155. The obtained S/N ratios were 16 for 2.5 pmol and 8.0 for 0.25 pmol. The ion images at *m*/*z* 4155 corresponding to the [M−H]^−^ of ASO-1 were reconstructed using FlexImaging. The ion images of the ASO-1 were obtained at 2.5 pmol and 0.25 pmol. LOD was calculated from the ratio of the laser irradiation area (6.25π pm^2^) and the area where the ion image of 0.25 pmol of ASO-1 was obtained (Fig. 1d) (LOD=0.25 pmol ×laser irradiation area/the area where the ion image of 0.25 pmol of ASO-1). Therefore, the LOD of ASO-1 by MALDI-IMS was 3.6 amol (SD=0.17). Similarly, the LOD of ASO-2 was also calculated. A peak at *m*/*z* 6683 corresponding to the [M−H]^−^ of ASO-2 was detected at 25 pmol (Fig. 1e) and 2.5 pmol (Fig. 1f). The obtained S/N ratios were 16 for 25 pmol and 11 for 2.5 pmol. The ion images of the ASO-2 were obtained at 25 pmol and 2.5 pmol. The LOD of ASO-2 was 35 amol (SD=1.7).

**Table 1:** Table 1. Sequences of ASO-1 and ASO-2.

	Sequence	Molecular weight	Exact mass
ASO-1	5′-GsCsAsTsTsGsGsTsAsTsTsCsA-3′	4157.37	4154.42
ASO-2	5′-GsCsGsTsTsTsGsCsTsCsTsTsCsTsTs CsTsTsGsCsG-3′	6682.35	6677.59

s; phosphorothioate

Three different volumes of ASO-1 and ASO-2 were deposited on an ITO-coated glass slide. Scale bar is 1 mm. Mass spectra are shown. S/N ratios were 15 (a), 8.0 (b), 16 (e), 11 (f) . Reconstructed ion image of ASO-1 (d) and ASO-2 (h).

### Development of MALDI-IMS analysis method of ASO-1 using tissue sections of mouse thigh muscle

**Figure 2:**
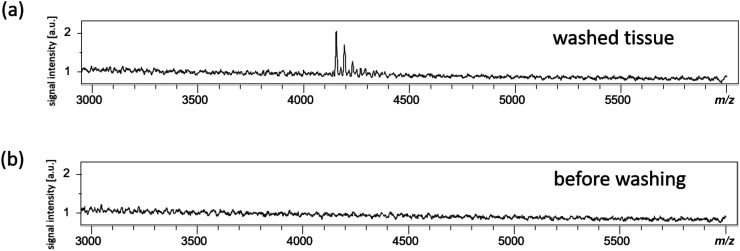
Fig. 2. Measurement of mouse muscle sections washed with organic solvents.

We administered ASO-1 to mouse thigh muscle and prepared tissue sections for IMS analysis. One microliter of the matrix solution was dropped onto the section, and IMS analysis was performed (mass spectra; Fig. 2, ion image; Fig. S1). A peak corresponding to [M−H]^−^ of ASO-1 could not be detected (Fig. 2b). Tissues contain a lot of low-molecular weight compounds such as lipids and metabolites, which suppress oligonucleotides. Therefore, to reduce ion suppression, we washed the tissue sections using an organic solvent. The [M−H]^−^ of the ASO-1 spectrum at *m*/*z* 4155 was obtained from the washed sections after dropping 1 μL of the matrix solution (Fig. 2a). The *m*/*z* of [M−2H+39]^−^ and [M−3H+78]^−^ were also detected.

Mass spectra obtained from an organic solvent-washed tissue (a) and a control tissue (b) sample.

**Figure 3:**
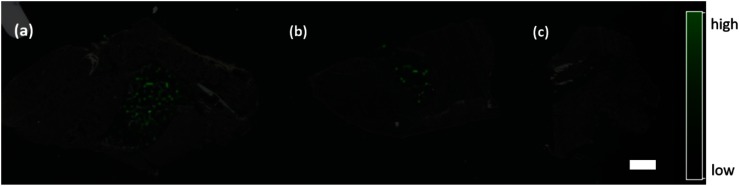
Fig. 3. The ion images of ASO-1 in mouse muscle sections.

Next, 50 and 5 nmol ASO-1 and control saline were administered to mouse thigh muscle tissue samples. After preparing the sections, a matrix solution was applied on the sections, followed by MALDI-IMS analysis (ion image, Fig. 3; mass spectra, Fig. S2). Consequently, ASO-1 signal was detected from the tissue sections to which ASO-1 was administered (Fig. 3a, b). Comparing the ion image of the tissue section administered with 50 nmol of ASO-1 (Fig. 3a) to that administered 5 nmol of ASO-1 (Fig. 3b) revealed that the signal intensity increased with the dose.

The section administered with 50 nmol (a), 5 nmol (b) ASO-1, and the control section (c). Scan pitch is 150 μm. The scale bar is 2 mm.

### MALDI-IMS analysis of ASO-2 in rat eyeball

**Figure 4:**
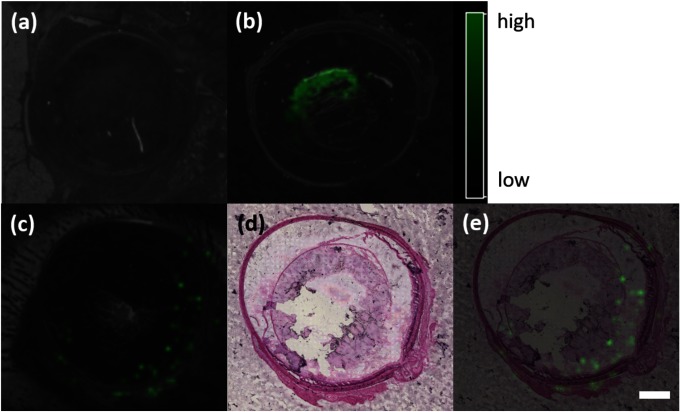
Fig. 4. The ion images of ASO-2 in rat eye sections.

We selected the sequence of fomivirsen (Vitravene^®^), which was the first approved oligonucleotide therapeutic product for cytomegalovirus retinopathy in AIDS patients in 1998. Eyeballs of rats were removed immediately and 30 min after ASO-2 administration. Tissue sections were prepared and analyzed by MALDI-IMS (ion image, Fig. 4; mass spectra, Fig. S3). The [M−H]^−^ of the ASO-2 spectrum at *m*/*z* 6683 was then obtained (Fig. S3). The ion images at *m*/*z* 6683 corresponding to ASO-2 were reconstructed. Consequently, in tissues excised immediately after ASO-2 administration, ASO-2 signal was localized in the central part of the vitreous body (Fig. 4b). In contrast, in tissues excised 30 min after ASO-2 administration, ASO-2 signal localized more in the peripheral part than in the central part of the eyeball (Fig. 4c). ASO-2 reached the retina as the action site 30 min after administration (Fig. 4e).

The tissue immediately (b), 30 min (c) after administration of ASO, and control section (a). The section stained with HE after measuring by IMS (d) and merged image of c and d (e). The scale bar is 2 mm. The scan pitch is 150 μm.

## DISCUSSION

Dosages of oligonucleotide therapeutics are in mg/kg-concentrations for antisense oligonucleotides, and in μg/kg-concentrations for siRNAs and miRNAs whose action mechanism is RNA interference.^34)^ Considering their very low concentrations, highly sensitive detection methods are required for pharmacokinetic analyses of oligonucleotide therapeutics. Therefore, in this study, we first investigated the sensitivity of MALDI-IMS for oligonucleotide measurements (Fig. 1). Based on our results, a concentration of as low as 3.6 amol of ASO-1 and 35 amol of ASO-2 on a glass slide could be measured, rendering the method highly sensitive. However, when detecting ASO-1 in tissue sections, the measurement sensitivity reduced by approximately 100 times (Fig. S4); nevertheless, femtomole amount could be detected. Thus, we believe that MALDI-IMS is suitable for investigating the pharmacokinetics of oligonucleotide therapeutics.

Many low-molecular weight compounds, such as lipids and metabolites, are more easily ionized in tissues than in oligonucleotides. As these substances interfere with the ionization of oligonucleotides, ASO-1 could be detected even by measuring the tissue section to which ASO-1 was administered (Fig. 2b). In this study, tissue sections were washed with an organic solvent to remove lipids, which are abundant in tissues. This technique has been previously used as the washing method for detecting proteins.^35)^ Because ASO-1 signal was detected in the sections washed using the organic solvent (Fig. 2a), it seems possible to reduce ion suppression by removing lipids *via* washing. The *m*/*z* of [M−2H+39]^−^ and [M−3H+78]^−^ were assumed to be potassium adduct ion. The salts were not completely removed by the washing process.

ASO-1 (50 or 5 nmol) was administered to the thigh muscle of mice, followed by IMS analysis. The signal intensity was higher from the tissue to which 50 nmol of ASO-1 was administered (Fig. 3). In MALDI-IMS analyses of oligonucleotides, relative quantitativeness was obtained.

Regarding the results of MALDI-IMS analyses of ASO-2 in rat eyeballs, only the peak corresponding to *m*/*z* 6683 of ASO-2 was detected, and no peak of the oligonucleotide decomposed by nucleases was detected (Fig. S3b). In this experiment, the phosphorothioate oligonucleotide was intravitreally administered, and the eyeball was removed after 30 min. Phosphorothioate oligonucleotides are several times more stable than natural oligonucleotides.^36)^ The vitreous body has lower levels of nucleases than blood. Thus, it was considered that oligonucleotides cleaved with a nuclease would be too small to be detected *via* mass spectrometry. In the past, as oligonucleotides were easily decomposed by a nuclease, topical oligonucleotide therapeutics that were not easily affected by decomposition have been developed. In recent years, due to the remarkable progress in the development of modified nucleic acids, resistance of oligonucleotides to nucleases and their stability in the body has greatly improved. Therefore, research on oligonucleotide therapeutics is progressing not only for topical administration but also for systemic administration. In 2013, mipomersen (Kynamro^®^), a drug for hypercholesterolemia, was approved for the first time as an oligonucleotide therapeutic for systemic administration,^37)^ and three more drugs for systemic administration were subsequently approved. Even with chemically modified oligonucleotides systemically administered, there is a possibility that the terminal nucleotide may be decomposed by a nuclease before reaching the target site. In such a case, an IMS-based method is required that can detect the parent drug and its metabolites simultaneously. The present study paves the way for future analysis on the distribution of administered oligonucleotides and their metabolites in tissues to support oligonucleotide therapeutics.

## CONCLUSION

We succeeded in the development of a new methodology for the analysis of oligonucleotides by MALDI-IMS. When phosphorothioate antisense oligonucleotide was administered into the eyeball of rats, the oligonucleotide reached the retina after 30 min without undergoing decomposition by nucleases.

## Notes

Mass Spectrom (Tokyo) 2018; 7(1): A0070
