# Supplementary material for: Distribution of Antisense Oligonucleotides in Rat Eyeballs Using MALDI Imaging Mass Spectrometry
Source: Mass Spectrom (Tokyo). 2018 Sep 11;7(1):A0070. doi: 10.5702/massspectrometry.A0070 (PMC6131115; doi:10.5702/massspectrometry.A0070)
Supplement: Supplementary file 1 [file massspectrometry-7-1-A0070-s001.pdf]

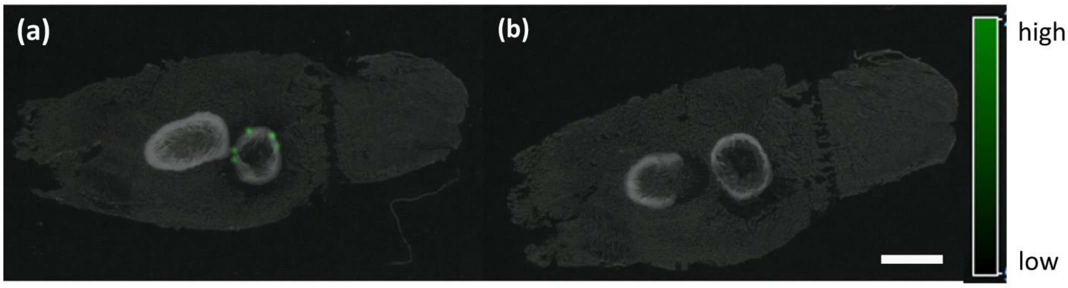

Figure S1. Measurement of mouse muscle sections washed with organic solvents. The ion image of ASO-1 was reconstructed from an organic solvent-washed tissue (a) and a before washing tissue (b) sample.

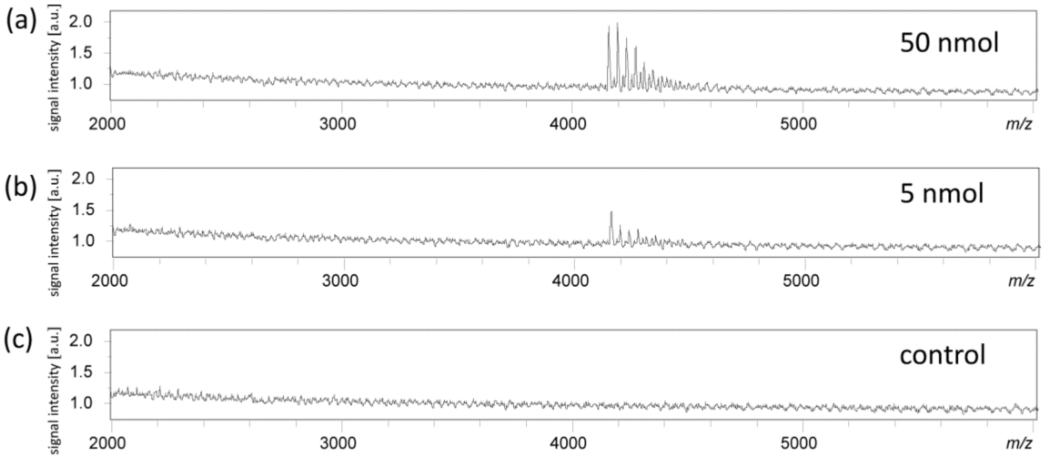

Figure S2. Measurement of mouse muscle sections. Mass spectra obtained from the sections administered with 50 nmol (a), 5 nmol (b) ASO-1, and control section (c).

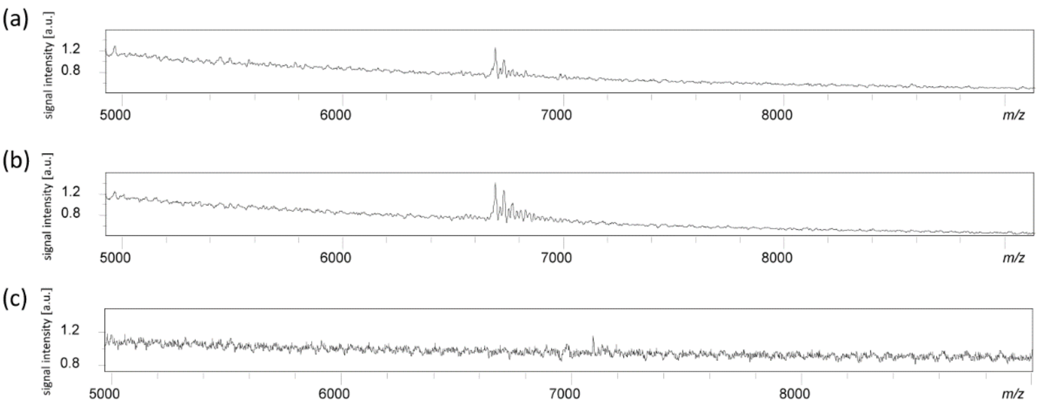

(d)

	Exact mass
ASO-2 (21 mer, administered drug)	6677.59
20 mer oligonucleotide (metabolite of ASO-2)	6332.56

Figure S3. Measurement of rat eye sections. Measurement of rat eye sections. Mass spectra obtained from the tissue immediately (a), 30 min (b) after administration of ASO-2, and control section (c). Exact masses of ASO-2 (21 mer) and single base decomposed oligonucleotide (20 mer) (d).

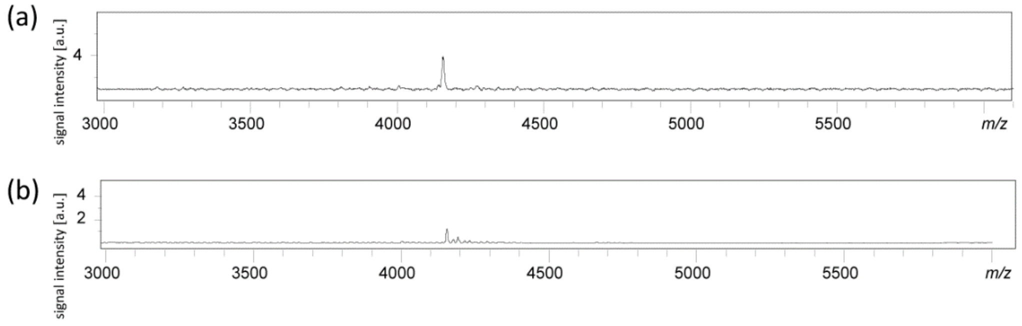

Figure S4. Comparison of signal intensities of 0.25 pmol ASO-1 on glass (a) and 25 pmol ASO-1 on tissue (b).
